# The “Double Bind” of Gender‐Based Violence: Secondary Victimization in Courtroom Cross‐Examinations

**DOI:** 10.1002/bsl.70025

**Published:** 2025-11-23

**Authors:** Selena Mariano

**Affiliations:** ^1^ Department of Political Sciences University of Perugia Perugia Italy

**Keywords:** Conversation Analysis, gender studies, gender‐based violence, male violence, Membership Categorization Analysis, secondary victimization

## Abstract

This paper examines how secondary victimization is interactionally produced during courtroom cross‐examinations of women who have experienced sexual violence. Drawing on Ethnomethodology, Conversation Analysis and Membership Categorization Analysis, the study investigates how defense attorneys invoke rape myths and gendered stereotypes to challenge victims' credibility and moral character. Using the extracts of two cross‐examinations from the celebrity trial *CA v. Winslow II* (2019), the results highlight how interactional features of questioning reproduce cultural assumptions that legitimate secondary victimization, constructing victims as unreliable or complicit. The findings highlight the “double bind” faced by women in sexual assault trials: they must appear both emotionally credible and rationally composed to be believed, yet any deviation from this ideal invites disbelief. Methodologically, the paper underscores the underutilized potential of EMCA in legal‐linguistic research to reveal how institutional talk reproduces gendered injustice through ordinary conversational practices.

## 1 Secondary Victimization: Defining the Phenomenon

Secondary victimization has been extensively documented within psychology, criminology, victimology, and by international institutions. As reconstructed by Pemberton and Mulder (2023), the first study to explicitly define this phenomenon (see also Wemmers 2013) was Symonds (1980). Symonds posited that secondary victimization comprised, from the victim's perspective, a perceived rejection and lack of expected support from the community, institutions, healthcare professionals, society, and even family or friends, directed towards an individual experiencing trauma or victimization. Given that victims of various crimes can be subjected to similar treatment, Williams (1984) introduced the concept of secondary victimization, defining it as the cumulative, prolonged, and exacerbated negative consequences arising from judgmental attitudes and behaviors, which manifest as a lack of support, condemnation, or marginalization.

Condry (2010, in Shoham et al. 2010) argues that crime victims experience secondary victimization when their condition is aggravated by the reactions of others and the treatment received within the criminal justice process. The author further emphasizes that this issue receives particular attention in studies concerning victims of sexual violence and harassment. In these contexts, victims frequently report feeling re‐traumatized when they report the crime, undergo invasive medical examinations and intensive interrogations, and, should the case proceed to court, face cross‐examination where their credibility is challenged (see also Madigan and Gamble 1991; Martin and Powell 1994).

On an institutional level, the Italian Parliamentary Commission of Inquiry on Femicide and All Forms of Gender‐Based Violence (hereafter simply “the Commission”), in its 2018 report, defines secondary victimization as a process through which a victim of male violence relives conditions of suffering similar to those already endured, often due to institutional procedures following a report or the initiation of legal proceedings. The Commission highlights that this phenomenon, particularly underestimated in gender‐based violence, discourages the reporting of crimes. Furthermore, it emphasizes that secondary victimization, like all forms of gender‐based violence, is deeply rooted in cultural norms: institutional representatives, as expressions of society, can unconsciously transmit gender biases and stereotypes that promote victim blaming.

Similarly, European institutions too have provided a definition of secondary victimization. Specifically, the Council of Europe, in the *Recommendation Rec(2023)2*, defines it as a form of victimization not directly linked to the criminal act but rather to the response of institutions and individuals towards the victim. The European Parliament and the Council, in the *Directive (EU) 2024/1385*, adopt a different perspective, observing that the use of evidence concerning past sexual behavior to undermine a victim's credibility or consent reinforces harmful stereotypes and generates further victimization. They therefore emphasize that, while respecting the right to defense, questions, investigations, and evidence related to a victim's past sexual life should not be permitted during investigations or trials.

In the U.S., the so‐called Rape Shield Law has been in place since 1994, with several amendments introduced over the years. Although this legislation was originally designed to protect complainants from intrusive and prejudicial questioning about their prior sexual history, it has faced sustained criticism for its limited effectiveness in preventing secondary victimization (Matoesian 1993, 1995). One major limitation lies in its narrow scope, which frequently excludes evidence relating to the complainant's prior sexual conduct with the defendant or other forms of evidence deemed “relevant” to consent. Such restrictions often leave victims exposed to credibility challenges based on their past behavior, effectively reproducing rape‐myth reasoning and re‐traumatizing victims during the legal process. The inconsistent implementation and enforcement of the Rape Shield Law across jurisdictions further undermines its intended protective function, resulting in uneven safeguards for victims nationwide, as secondary victimization continues to manifest in courtroom interactions largely without objection.

In this vein, Craig (2021) describes secondary victimization as a *symbolic assault* on the complainant, perpetrated through defensive strategies that include humiliating or excessively lengthy cross‐examinations, specious requests for private documentation, and the deployment of stereotypes regarding consent, communication, attire, revenge, marriage, past sexual history, therapeutic paths, absence of resistance, and delays in reporting. Consequently, Craig stresses that for many victims, the judicial process can be as traumatic as the assault itself, impeding their recovery and re‐appropriation of their bodies and well‐being. Similarly, Pemberton and Mulder (2023) observe that, in most cases, secondary victimization does not occur in violation of existing criminal law but rather constitutes a consequence of its very operation.

A different, and more compelling, way to define secondary victimization is to look at the defense's interactional stance: namely, using legal definitions of consent based on a male perspective to pressure the victim into justifying any ambiguous signals (Conley and O’Barr 2005). Resistance is evaluated based on male standards; discrepancies in testimony are amplified to suggest unreliability or falsehood; and post‐rape behavior is scrutinized, especially when it does not conform to cultural expectations of an “appropriate” reaction. Such definition is further emphasized by Gunby and Carline's (2020) exploration of barristers' emotional and strategic engagement in rape trials, thereby extending the focus to how cross‐examination sequences reproduce myth‐based presumptions and contribute to secondary victimization.

In conclusion, secondary victimization appears in literature as a well‐known phenomenon, not ignored even by institutions. Therefore, it is imperative to identify alternative and effective strategies to reduce this phenomenon. To this end, it is first necessary to know how to recognize it, starting with identifying its most evident forms, namely rape myths and gendered stereotypes, as they emerge in cross‐examinations of victims of male violence.

### 1.1 Rape Myths and Gendered Stereotypes

The expression “rape myths” was introduced in the 1970s by Brownmiller (1975) and Estrich (1976), referring to a set of cultural assumptions about sexual violence and what is recognized as a “real rape” (Smith 2018). Bohner et al. (1998, in Smith 2018) define these myths as prescriptive or descriptive beliefs about sexual violence that serve to deny, minimize, or justify the act itself, conveying the idea that all victims react uniformly to rape. Burrowes (2013) argues that these myths are employed to negatively judge the actions of victims or survivors, ignoring the role that emotions such as fear, guilt, and shock play in decision‐making processes.

These beliefs remain deeply ingrained in contemporary culture and include, in addition to the definition of “real rape,” behavioral prescriptions for how a victim should act before, during, and after an assault, who should be identified as the assailant, and how the crime should be committed (Temkin and Krahé 2008). These myths are then upheld by professionals involved in the criminal justice system, thus contributing to secondary victimization, which manifests through blaming, questioning credibility, and obstructing access to justice (Smith and Skinner 2017).

According to Smith (2018), Bohner et al. (2009) identify four main categories of rape myths, derived from analyses of academic literature and NGO surveys: those that blame the victim, question the accusation, justify the perpetrator, and restrict rape to certain social contexts. Various studies have highlighted the concrete impact of these beliefs in legal contexts. Dinos et al. (2015) observe that attorneys' use of myths can influence trial outcomes, with a greater influence in Europe than in the United States. Ellison and Munro (2009) demonstrate that the defense's adoption of these myths reduces victim credibility. Finally, Rose et al. (2006) note that delays in reporting or inconsistencies in testimony tend to be interpreted by juries as signs of deceit.

Gendered stereotypes represent a different form of secondary victimization, particularly in courts, where defense attorneys utilize a victim's gender to undermine their credibility (Smith 2018). For the purposes of this paper, the definition proposed by Smith (2018) is adopted, which posits that gendered stereotypes consist of inferences based on common cultural representations of gender. From the identification of gender, jurors are purportedly induced to infer further characteristics, such as unreliability (see Schneider 2004). Various studies have contributed to understanding the effects of gender stereotypes in trials. Fenton (1998), for example, showed that lawyers use narratives infused with stereotypes, like that women are inherently emotional and irrational, to push jurors towards a quick decision. This stereotype, according to Brescoll (2016), represents a dominant organizing principle in gender beliefs across many cultures. Shields (2002) considers it one of the most deeply rooted stereotypes in Western societies, founded on the dichotomy of woman‐emotional/man‐rational. Bernau (2007) highlights a further dichotomy between the figure of the virgin and the prostitute, which gives rise to a mutually exclusive representation of female sexual identity: prostitutes are negatively connoted as irrational subjects, while virgins are associated with positive qualities such as purity, passivity, and fragility. However, even a “pure” woman, as Taslitz (1999) observes, is not necessarily deemed reliable in court.

Considering gendered stereotypes in secondary victimization is essential for understanding its scope. Milivojevic and Ćopić (2010, in Shoham et al. 2010) observe that the link between female victimization and social expectations related to femininity generates the idea that “appropriate” female behavior constitutes a fundamental tool for avoiding violence. It is from this premise that the process of victim blaming originates.

A noticeable feature of these revictimizing strategies is that they are enacted by actors within the courtroom through the sole instrument at their disposal: language—in the form of questions, judgments, and so forth. Central, therefore, is the understanding of such role in the mechanisms of secondary victimization. Thus, the objective of the present work is to analyze how, during legal proceedings, the interaction unfolds between defense attorneys and victims, and consequently secondary victimization takes shape and is perpetrated against victims of male violence. This is done, specifically, by looking at how rape myths and gendered stereotypes, and their relative implications, are interactionally built and delivered by defense attorneys in cross‐examinations and, ultimately, how victims resist to such interactional structures. Differently than the studies previously cited, therefore, the analytical focus of this work shifts the perspective of secondary victimization from the perception of the victims and from the results of rape myths in verdicts, to look at how and when this happens interactionally during the legal proceeding (Hudspith et al. 2024).

In order to do so, two cross‐examinations from the celebrity trial *CA v. Winslow II* (2019) are analyzed, in the present work, with an Ethnomethodological (EM) approach, particularly through Conversation Analysis (CA) and Membership Categorization Analysis (MCA). The approach, as the techniques, are the most suitable to appreciate not simply the structure of the language used, but specifically the smallest nuances of the interaction as it unfolds, turn by turn. By focusing on natural occurring data, it is possible to observe the fine grain of social interaction, how the ordinary methods used in daily life are applied in institutional interactions and the effects of these methods in the construction of a social order that transforms victims of male violence in second‐tier victims.

## 2 Methodology

As previously introduced, central in courtroom secondary victimization is how the interaction unfolds: specifically, are the attorneys' turns of questions that, as they unfold, reveal a discriminatory strategy towards women victims of gender‐based violence. Examining witnesses is necessary for the parties to fulfill their burden of proof which requires, more so for the prosecution than the defense, to demonstrate the validity of their case (Grilli 1998). It is therefore a place where witness‐produced evidence is formed through dialog and the testimony of various individuals (Goodwin 1994).

Thus, it is only natural to consider language as a central tool within the burden of proof, particularly focusing on how the evidence created within the trial is not only that which verifies the facts of the crime, but is also evidence in favor of a moral judgment regarding the victims, who find themselves discredited due to their private lives, their choices, or their sexual history and consequently revictimized. The aim of this research, therefore, is not to identify wrong convictions, or to search quantitatively for a trend, but rather to look in detail how *language in interaction* generates actions with discriminatory potential.

In this framework, it is of the utmost importance to consider Drew's position that social conduct and relations constitute accountable phenomena, constructed by “reporting, describing, and reasoning,” addressing “the central role that language plays in constructing social reality” (Drew 1998). Moreover, Drew argues that “any consideration of the accountability of social conduct brings directly into focus moral dimensions of language use” (Drew 1998). Indeed, according to Stokoe, the moral order and values are in “participants' descriptions of activities and what they treat as legitimate or illegitimate warrants for certain sorts of action ascriptions” (Stokoe 2003).

Considering this premise, the approach used in this research is Ethnomethodology (EM), operationalized through Conversation Analysis (CA) and Membership Categorization Analysis (MCA) (Clayman and Maynard 1995; Hester and Eglin 1997).

EM, founded by Harold Garfinkel, studies the methods people use to create and maintain social order in everyday life. Rooted in phenomenology (especially Alfred Schütz) and Wittgenstein's philosophy of language, it departs from traditional sociology by focusing on how people produce meaning and order through interaction, rather than treating society as a pre‐existing structure (Garfinkel 1967). Its methodological principles include: (i) studying naturally occurring interactions without manipulation; (ii) observing members' methods (*ethno‐methods*) as the real data of sociology; (iii) holding unique adequacy, meaning researchers must understand and describe phenomena using participants' own methods; (iv) to adopt ethnomethodological indifference, meaning that researchers describe, not judge, the phenomena they observe, focusing on visible, ordinary actions as evidence of how society is “done” (Garfinkel 1967). In essence, EM shows that social order is locally produced, visible, and reflexive, that is constructed and maintained through the very practices that display it (Cora Garcia 2023).

Developed by Garfinkel's student Harvey Sacks, Conversation Analysis (CA) studies the detailed organization of talk in interaction. It is empirical and inductive, based on recordings of natural speech and on the pillar that conversation is not random, but governed by an internal order that can be analyzed (Sacks 1992). Given this, language is considered as social action (e.g., requesting, inviting) rather than merely transmitting information, and its meaning depends on the local context and sequential positioning of utterances. Furthermore, in CA, the analysis must reflect how participants themselves understand and respond to each other. Finally, some interactional rules have been identified: (i) recipient design, namely speakers tailor utterances to specific listeners; (ii) local and interactional management, that is participants co‐manage the flow of turns; (iii) sequentiality, indicating that each conversational turn gains meaning from its position in the sequence; (iv) sequentiality is the foundation of what has been identified as a turn‐taking system, that is conversation follows an implicit “one speaker at a time” rule with smooth transitions and repair mechanisms; (v) some of these turns can be structured into adjacency pairs, basic paired actions (e.g., question‐answer, greeting‐greeting, etc.) that structure talk (Sacks et al. 1974). CA thus exposes the micro‐organization of talk as the foundation of social interaction.

Membership Categorization Analysis (MCA), also developed by Sacks, explores how people use categories (e.g., “mother,” “child,” “doctor”) in talk to construct social meaning. Categorization is too considered a practical activity, or even a tool people use to describe, explain, or evaluate others and events (Sacks 1972). Categories, in this framework, have specific characteristics. First, they are inference‐rich, which means they carry shared social knowledge. Second, categories cluster into Membership Categorization Devices (MCDs), such as “family” or “profession”; even if a category falls into a MCD, though, it can also be part of another one. Categories also have rules of application: (i) the economy rule, participants choose the most relevant category for the situation; (ii) the consistency rule, that is once a category from a collection is used, others from the same collection apply; (iii) the hearer's maxim, “if two or more categories are used to categorize two or more members of some population, and those categories can be heard as categories from the same collection, then: hear them that way” (Sacks et al. 1974, 219–220, as in Cora Garcia 2023, 157); (iv) the viewer's maxim, “if a member sees a category‐bound activity being done, then, if one sees it being done by a member of a category to which the activity is bound then see it that way” (Sacks 1972). Thus, MCA reveals how categorization shapes social understanding and moral judgment in everyday and institutional interactions, showing that language both reflects and constructs social order (Fitzgerald and Housley 2015).

While early research primarily examined everyday conversation, their application to institutional contexts, particularly the courtroom, has generated profound insights into how law, morality, and authority are discursively constructed. Within the legal domain, EMCA have illuminated the methods through which participants organize talk, make sense of institutional norms, and collaboratively produce the appearance of objectivity and justice.

EM, with its emphasis on the methods used by members in interaction, has long been concerned with how legal professionals accomplish their work in practice. From Garfinkel's (1967) seminal analysis of jury deliberations to later studies (see Maynard and Manzo 1993), scholars have demonstrated that even within the highly formalized structure of the courtroom, judges, attorneys, and jurors rely on practical reasoning and common sense to interpret laws, apply procedural rules, and construct verdicts. This approach gave rise to the influential “studies of work” tradition, which investigates the situated practices of legal professionals, their reasoning methods, evidentiary procedures, and interpretative strategies through ethnographic observation and detailed discourse analysis (Travers and Manzo 1997; Dupret and Lynch 2015). EM research thus reframes the courtroom as a site of local order production, where justice is not merely administered but interactively achieved through talk and practical judgment.

Building on this foundation, CA has examined courtroom interaction as a distinct type of institutional talk (Atkinson and Drew 1979; Heritage 2004). CA studies reveal that institutional interactions are characterized by their own sets of constraints, goals, and asymmetries. The courtroom exemplifies this through its pre‐allocated turn‐taking system, rigidly defining who can speak, when, and in what capacity. Attorneys, for instance, control the questioning process, object, and make statements, whereas witnesses primarily respond within the confines of prescribed question‐answer adjacency pairs (Heritage 2004). These asymmetries are reflected in the design of turns and the conditional relevance between questions and answers (Schegloff and Sacks 1973). CA has thus provided a systematic account of courtroom discourse, analyzing the micro‐sequential organization of talk and its role in shaping credibility, accountability, and persuasion.

Although EMCA have profoundly advanced our understanding of courtroom interactions, research has only recently begun to address how these interactional practices contribute to secondary victimization. Scholars such as Hildebrand‐Edgar and Ehrlich (2017) and Matoesian (1993, 1995) have highlighted the “double bind” faced by women victims of sexual violence, who must simultaneously demonstrate credibility while navigating a discursive environment structured by skepticism and gendered norms. A recent contribution by Richardson et al. (2025) applies CA and Discursive Psychology to examine how rape myths are reproduced and negotiated during jury deliberations in mock rape trials. Their analysis identifies “contrastive discrediting devices,” that is, interactional practices through which jurors contrast a complainant's behavior with culturally expected notions of how a “real” victim should act. By treating rape myths as interactional resources rather than fixed beliefs, the authors show how common‐sense reasoning and moral assumptions shape collective decision‐making. A complementary study by Tennent and Richardson (2025) apply CA and Discursive Psychology to examine police calls related to domestic violence during COVID‐19 lockdowns in the United Kingdom and New Zealand. Analyzing 200 emergency and non‐emergency calls, they show how callers and call‐takers jointly construct the moral and spatial order of “home,” transforming everyday actions into matters of accountability and police intervention. Their findings reveal how institutional responses to domestic violence are interactionally produced, illustrating how communication practices can both facilitate and constrain victims' access to justice.

Nonetheless, the systematic study of secondary victimization as an interactional phenomenon remains underdeveloped. This thesis aims to fill that gap by applying an EMCA‐informed lens to explore how courtroom talk can reproduce harm, bias, and inequality, thus revealing the linguistic mechanisms through which justice itself is socially and morally accomplished.

## 3 Data Overview

The data presented in this work comes from a collection of 12 cross‐examinations, that constitute the moment for the defense in a trial to examine the prosecution's witnesses, of which the victim(s) of a crime are part of.

Of such cross‐examinations, seven are from Italian courts, and five are from the U.S. celebrity trial *CA v. Winslow II* (2019), in which former pro‐football player Kellen Winslow II was accused of various grades of assault to the detriment of five different women. Whilst the Italian data was not recorded, hence the only data available are the courtroom transcripts, *CA v. Winslow II*'s video recordings are available on YouTube, for a total of circa 13 h of cross‐examinations. These have been transcribed by the author according to EMCA's methodology using Jeffersonian conventions, reported at the end of the present paragraph (Table 1). The availability of video recordings, and therefore the closer alignment of this data with the EMCA methodology, has made them a more suitable source for illustrating the phenomenon of secondary victimization through the sequential construction of rape myths and gendered stereotypes. Of five cross‐examinations, only a total of four excerpts of two of them, amounting to circa 3 min, have been selected for the present work, as they better exemplify the defense's work of constructing rape myths and gendered stereotypes through the question‐answer sequence of the courtroom turn‐taking system.

**TABLE 1:** Jeffersonian transcript conventions.

Symbol	Definition and use
[yeah]	Overlapping talk
[okay]	
=	End of one TCU and beginning of next begin with no gap/pause in between (sometimes a slight overlap if there is speaker change). Can also be used when TCU continues on new line in transcript
(.)	Brief interval, usually between 0.08 and 0.2 s
(1.4)	Time (in absolute seconds) between end of a word and beginning of next
Word Wo:rd	Underlining indicates emphasis. Placement indicates which syllable(s) are emphasized
	Placement within word may also indicate timing/direction of pitch movement (later underlining may indicate location of pitch movement)
wo::rd	Colon indicates prolonged vowel or consonant
	One or two colons common, three or more colons only in extreme cases
↑word	Marked shift in pitch, up (↑) or down (↓). Double arrows can be used with extreme pitch shifts
↓word	
.,_¿?	Markers of final pitch direction at TCU boundary:
	Final falling intonation (.)
	Slight rising intonation (,)
	Level/flat intonation (_)
	Medium (falling‐)rising intonation (¿) (a dip and a rise)
	Sharp rising intonation (?)
WORD	Upper case indicates syllables or words louder than surrounding speech by the same speaker
word‐	A dash indicates a cut‐off. In phonetic terms this is typically a glottal stop
>word<	Right/left carats indicate increased speaking rate (speeding up)
<word>	Left/right carats indicate decreased speaking rate (slowing down)
.hhh	Inbreath. Three letters indicate “normal” duration. Longer or shorter inbreaths indicated with fewer or more letters
hhh	Outbreath. Three letters indicate “normal” duration. Longer or shorter inbreaths indicated with fewer or more letters
£word£	Pound sign indicates smiley voice, or suppressed laughter
(word)	Parentheses indicate uncertain word; no plausible candidate if empty
(( ))	Double parentheses contain analyst comments or descriptions

The analytical procedure was carried out following these steps: using MCA, a thematic search was conducted within the defense attorneys' turns to identify instances in which rape myths and/or gendered stereotypes, as classified by Smith (2018), were invoked. Subsequently, the victims' responses to such prompts were examined from a CA standpoint, focusing only on cases that displayed signs of resistance, such as raising their voice, pauses or silences before responding, the use of irony, or the production of alternative accounts to reformulate the descriptions of their actions proposed by the attorneys, to adopt a participant's perspective and look at credibility attacks only where the complainants showed them as such. This mechanism, namely, attorneys asking questions that sequentially construct a narrative infused with rape myths and gendered stereotypes to discredit the victim, and the victims' resistance despite being constrained to respond almost exclusively to yes/no questions, emerged as the most evident form of secondary victimization in the data collected. Such interactional scaffolding has led the victims in the extracts to face a “double bind” (Matoesian 1993, 1995): on the one hand, they face the interactional constraints that the courtroom setting imposes; on the other, they have the need to defend themselves from the repeated attacks to their credibility.

Given the sensitive nature of the data analyzed, particular attention was paid to the ethical management of both the materials and the interpretive process. Although the courtroom data examined in this study are part of the public record, they contain highly personal and distressing accounts. Accordingly, all identifiable information, including names and personal references, has been anonymized. The pseudonyms “Jane Doe 1” and “Jane Doe 2” are used throughout, following established conventions in EMCA and socio‐legal research on sexual violence.

A central ethical dilemma of this work lies in the dual status of the data: while legally public, their re‐use for analytical purposes risks reproducing the very exposure and vulnerability that secondary victimization entails. To mitigate this, excerpts were selected not for their sensational or emotional impact but for their analytical relevance to the reproduction of gendered stereotypes and rape myths. The presentation of data thus aims to balance empirical rigor with respect for participants' dignity and privacy. Ultimately, the collection has been gathered during the author's PhD research, and the ethical approval was granted by the author's thesis supervisor.

Finally, a methodological note on transcripts is needed. As Richardson (2024) highlights, courtroom transcripts are not neutral records of spoken interaction but entextualizations, meaning subjective textual reconstructions shaped by those who produce them, thus emphasizing how transcription choices affect the representation of meaning. For EMCA research, this perspective underscores the need for reflexivity and methodological transparency: transcripts must be treated as interpretive artifacts rather than verbatim reproductions of talk. Recognizing this allows researchers to better account for how interactional details, such as pauses, intonation, and sequencing, shape the construction of credibility and accountability in courtroom discourse.

## 4 Secondary Victimization in Courtroom Cross‐Examination

In the current section, the data analyzed shows that whilst the complainants find themselves managing attacks to their credibility, conducted by the defense attorney during cross‐examination as explained in the previous paragraph, they also face a “double‐bind”: linguistic and institutional norms force them into contradictory expectations of femininity and credibility, ensuring that any self‐presentation can be discredited (Matoesian 1993, 1995).

As aforementioned, the following excerpts come from the celebrity trial *CA v. Winslow II* and feature the cross‐examination of two of the five victims (Jane Doe 1 and 2, respectively, abbreviated as JD1 and JD2). Winslow's defense counsel is reported in short form as DC, as well as PA for the prosecution attorney. Finally, when present, J stands for the judge.

Consider Jane Doe 1 (JD1). According to her report, at the time of the crime, that happened in Encinitas (San Diego, CA) in 2018, she was in her early fifties, unemployed, and lived with her mother. On the morning of St. Patrick's Day 2018, she went on a walk. Eventually, she got tired and decided to hitchhike. In that moment, the defendant stopped and asked her where she needed to go. JD1 replied that she was directed towards Vulcan Avenue, and the defendant agreed to give her a ride. Once in the car, he introduced himself as “Dominique”—identified by the complainant as Kellen Winslow II—and they chatted about St. Patrick's Day. After a while, the defendant said to JD1 that he would not take her to Vulcan Avenue, rather he said to her that he was going to rape her—menacing of killing her if she did not comply. Alas, the crime happened in a parking lot of a shopping area.

The first extract is nearly at the end of the cross‐examination. Here the defense attorney (DC) is inquiring about JD1 screaming and yelling after “Dominique” only when he drove away in his car, after committing the crime. The aim of DC is to create an ambiguity between a prior testimony of JD1, where she stated that she was too afraid to scream for help or to try to run away, and the fact that she did scream and run after the crime. This excerpt illustrates how the defense initiates a strategy of undermining the complainant's credibility through subtle appeals to rape myths and contradictions in her behavior. The cross‐examination begins by questioning Jane Doe 1's reactions during and after the assault, framing her conduct in a way that implies inconsistency with that of a “real” victim.

Extract 1 **“CA v. Kellen Winslow II”, Jane Doe 1 (JD1) cross‐examination by defence counsel (DC).**

1
 1 DC **NOW** you're screaming and yelling. (.) **correct?**=
 2 JD1  =Well he was already halfway go:ne¿ he was gone leaving.=
 3 DC =**Right** (that was the **point** is‐) **<you ran after the car> after** you were
 4    (.) **((JD1 coughs))** told to get the **fuck** out of the
 5    vehi[cle (.) (see) (.) >**let me ask you this**<]
 6 JD1    [HE WAS A::L: (.)]°[okay go °     ]
 7 DC After you were **to:ld** to get the **fuck** out of the vehicle (.) which you
 8    **did** >and the car starts taking off< **y↑ou now** for the **first time**, (.) 
 9    start **runni**↑**ng** after the **car**. (.) **correct?**
10    (1.9)
11 JD1 No i didn't run after the ca:r.(.) i just r:run to t‐the building to
12    see how‐which way he was going.=
13 DC =Okay so you ran to the building to see which way he was going you **ran**
14    that's the point (.) you **£d(h) id run£ right?**=
15 JD1  =RIGHT=

In line 1, DC poses a question opening with “now,” stressed both in emphasis and volume, making available the inference that the timing of the “screaming and yelling” is important. Then, the question is closed by the polarizing particle “correct.” Such particle suggests only two possible answers (yes or no), of which one is preferred. According to Pomerantz (1984), the preference mechanism organizes conversation so that socially affiliative responses like agreement are delivered promptly and directly, while dispreferred ones like disagreement are delayed or mitigated to preserve interactional harmony. In the case of JD1, given the affirmative polarization, the preferred answer would be “yes” (Pomerantz 1984). JD1, in line 2, replies, without giving such answer, with an account (i.e., an explanation) of why she was acting as such in that moment, that is because “Dominique” was now far, thus implying that “now” she was safe. The fact that she avoids completing the yes/no question posed by DC suggests that she can hear an accusation in DC's turn, therefore by giving the preferred answer she would agree to it, thus accepting the implicit accusation (Drew 1979). In lines 3 to 5, DC opens his turn with “right,” showing that he understood not only what JD1 said, but also that she correctly anticipated the accusation (“that was the point”), subsequently expanding it (“you ran after the car after you were told to get the fuck out of the vehicle see”). An overlap happens, which DC immediately corrects (“let me ask you this”), correction that JD1 accepts (“okay go”). Then, in lines 7 to 9, DC continues the correction by repeating the last part of his previous turn before the interruption (“after you were told to get the fuck out of the vehicle”), here without asking for confirmation (“which you did”). Then, DC continues his turn marking once again the timing of the action (“you now for the first time”), noticeable also from the emphasis in the intonation, and lastly concludes his accusation in a polarized form (“start running after the car correct”). It follows a 1.9 s pause, signaling the upcoming of a dispreferred answer (Pomerantz 1984), which opens JD1's turn (“no,” line 11), expanded by another account. Subsequently, DC exhibits once again his comprehension (“okay so you run to the building to see which way he was going,” line 13), and concludes his turn with the explicit accusation in a polarized form (“you ran that's the point you did run right,” lines 13 and 14), marked even more by the laughter through which he utters “you did run,” and such creating irony in his strategy (Clift 1999) to highlight the contradiction that when in danger JD1 did not run to save herself, when she had the occasion, but “now” that there is no more danger, she runs.

What is immediately available is the frequency of the various formulations of the questions about JD1 running—firstly after the car, then just running. One way of seeing these questions is as “conducive” ones: « (…) conducive questioning is a pragmatic discourse tool to enable barristers to control the testimony of witnesses. (…) it is primed to produce the kind of answers that barristers intend to hear from witnesses under examination» (Aina and Anowu 2023). But, even more importantly, DC is using these conducive questions to reinforce his accusation rooted in victim blaming rape myths. What the defense is referring to, indeed, is that there had been a moment before the crime happened, when JD1 could run. In that occasion, she did not run to save herself, thus “she did not act as a victim should,” which is a common rape myth, since a “true victim fights in anyway she can to resist the rape” (Ellison and Munro 2013). Therefore, according to DC's strategy, JD1 is not a real victim, and the crime has never occurred (otherwise she would have behaved like a victim). JD1, on the other hand, can hear this accusation and tries to manage it with traditional resources already documented in literature (Drew 1979), for example: avoiding attributing self‐blame, giving accounts, raising her voice (line 6), or taking long pauses before giving a dispreferred answer. What is noticeable absent, though, is the intervention of either the prosecution attorney or the judge to interrupt such derogatory line of questioning, in which JD1 is only culpable of not having avoided becoming a victim of rape.

While this first excerpt focuses on discrediting Jane Doe 1 through the rape myth that “real victims resist,” the next one expands this strategy. Here, the defense uses a broader set of rape myths, linking wealth, fame, and delayed reporting, to further erode her credibility and moral standing. By repeatedly invoking the defendant's status and the complainant's post‐rape behavior, the attorney reinforces cultural assumptions that associate victims' worth and truthfulness with their social and moral identity.

Extract 2 **“CA v. Kellen Winslow II”, Jane Doe 1 (JD1) cross‐examination by defence counsel (DC).**

1
 1 DC  So: you know that (.) so we know that (.) this individual
 2     >picked you up< in a **very nice car**, (.) °literally° **the**
 3     **car's worth some money**, (.) right?
 4      (0.9)
 5 JD1  £Right£=
 6 DC   =You say that he raped you?
 7      (.)
 8 JD1   Yes?=
 9 DC   =>**You waited a couple days, before you reported it?**<=
10 JD1  =Yes,=
11 DC   =>**You refuse giving the sart exam, or cooperating to further**
12     **investigation**, correct?<=
13 JD1  =Yes,=
14 DC   =A::nd **then you: sa::w after the arrest that he wa:s a pro**
15     **football player** >because **you looked at him, (.) on the**
16     **news**,< right?
17     (0.8)
18 JD1  Yes.=
19 DC   =And you s↑aw that he was k↑ellen w↑insl↑ow (.) **w↑insl↑ow's**
20     **a big name isn't it?**
21     (1.1)
22 JD1   [Y]↑[es]=
23 DC  [Yes?]
24 JD1   =°okay°=
25 DC  =An:d you also testified, (.) **that you WALK around, you cruise**
26     **around, and you talk to everybody (.) you talk to people on**
27     **the streets**. (.) correct?
28 JD1  Yes. ((sounds annoyed))=
29 DC  =**And did you talk to any LAWYERS**?
30      (0.9)
31 JD1  No. **((sounds annoyed))**
32 DC    Never talked to any lawyers at all.
33     (1.7)
34 JD1  **About money?**
35 DC   Yes (.) [ ab]out money
36 JD1      [No.]
37 DC   No? (.) okay.
38     (1.6)
39 DC    **None of your fr↑iends told you that you could °potentially°**
40     **get money out of this**.
41     (2.7)
42 JD1   No.=
43 DC    =And you're certain of THAT?=
44 JD1   =I'm p↑ositive_=

This excerpt comes from the end of the cross‐examination. In this moment it is common for attorneys to wrap up the salient points of the interrogation and sum up. It is standard practice to do so, because being the last thing in the witness examination it will probably be the easier part of it to recall (being timewise more recent than other parts). There is nothing different here in DC's turns than before: questions, polarization, and the stress in the intonation or the extension of letters to grasp the hearer's attention and focus it on specific subjects. The rhetorical effect is strong: a narrative, blaming JD1, almost never contradicted by the complainant, since she is constrained in very essential answers. This last feature is created by DC's turns, which require a very short yes/no answer, and at the same time are exploited by DC himself to keep a fast pace in his questioning, not giving JD1 time to expand her turn given the asymmetry in institutional talk (Heritage and Clayman 2010). In doing so, the attorney also uses strategically the repetition of the structure, that has already been proven to solicit skepticism (Pomerantz 1988; Matoesian 1995). But what is more important in this extract is that DC's turns do not involve at any moment the confirmation of the details of the crime. Rather, he inquiries about JD1's actions and intentions, leveraging different rape myths in each turn. In fact, JD1 is presented as belonging to the category “victim,” and this presentation is challenged by DC by soliciting rape myths through a contrast between “what a victim should do” and what JD1 does.

For example, being “picked up” in a “nice car” “worth some money” (lines 1–3) is not a category‐bound activity of a rape victim, rather, according to the viewer's maxim (Sacks 1972), is it possible to see JD1 as a sex worker. The other category‐bound activities pertaining to sex workers, used in lines 25–27 (“you walk around, you cruise around […] you talk to people on the streets”), solicit the inference that JD1 is a sex worker. But, as Silver et al. (2015) have found out, a common myth is that people working in prostitution cannot be raped. Moreover, waiting “a couple of days” before reporting (line 9), is not a category‐bound activity of a rape victim, but falls under reasons of suspicion and non‐credibility according to rape myths (delay in reporting is suspicious, Carr et al. 2014). Refusing to give a SART exam (Suspected Abuse Response Team, an evidentiary medical forensic exam) or “cooperating” (lines 11 and 12) is too seen as suspicious, because is thought that any victim of a crime should do anything they can to prosecute, and this can leverage the image of a false accusation (Lees 1997). Finally, mentioning the money and the fame of the accused can cast doubts on JD1's accusation, inferring that they are false allegations of a person looking to gain some money from this (CPS, 2013).

In conclusion, here too JD1 is accused of either not being a victim—having fabricated the accusations—or not being an “ideal victim”—accomplice of the crime she suffered. Her actions before, during and after her rape, are placed under high scrutiny and morally evaluated shifting the accusation on her individual character, rather than trying to establish how or whether the crime took place. Nonetheless, she employs the only action she can do (i.e., answering the questions) to create alternative description of what the attorney is trying to establish as a fact, in order to protect her credibility.

The interactional logic that underpins Jane Doe 1's questioning recurs in the examination of Jane Doe 2, where similar strategies are used to reframe her actions as morally suspect. Despite differences in background and circumstance, both cross‐examinations reveal a consistent defense tactic: constructing alternative moral categories to reassign blame to the victim.

Jane Doe 2 (JD2) shares a similar fate. At the time of the crime, in 2018, according to her recollection of events, she was 58 years old, experiencing homelessness and unemployment. She recounted that approximately 6 months before the rape, while on the street with numerous bags, she was approached by a man named “Kevin,” whom she later identified as Kellen Winslow II. He offered her a ride, which JD2 accepted, asking to be driven to the shelter where she was then residing. This initial interaction was followed by six or seven seemingly friendly encounters. Eventually, “Kevin” approached JD2, who had just lent her phone to a friend, inviting JD2 for coffee, to which the she agreed. In this occasion, the defendant drove to an isolated location and raped JD2.

The following extract demonstrates how these same mechanisms are applied to Jane Doe 1 are used against Jane Doe 2, whose socioeconomic vulnerability becomes a discursive resource for further discrediting her. Here, the defense's questions again rely on rape myths, particularly those linking sexual violence to motives of financial gain, and recast her as complicit rather than victimized.

Extract 3 **“CA v. Kellen Winslow II”, Jane Doe 2 (JD2) cross‐examination by defence counsel (DC).**

1
1 DC    =Understood (.) but↑ (.) **a gu:y, (.) in a super nice hummer,**
2       **(.) rolls up, a:nd you (like/what) thalk to the guy.** right?
3      (1.0)
4 JD2     Uh because **he seems like a friend not because of the £hummer£** hh=

DC's turn opens with an exhibit (“understood,” line 1), followed by a brief pause and a “but,” signaling disagreement. Then, the turn is expanded with a description of what was happening in that moment (“a guy in a super nice hummer rolls up and you like/what talk to the guy,” lines 1 and 2), formulated as a polar question (“right”). JD2 is supposed to give the preferred answer (Raymond 2003), in this instance a “yes,” but—as it is noticeable by the pause in line 3—JD2 completes the adjacency pair in a non‐conforming way, giving an alternative description, hearing an accusation in DC's turn. In fact, what DC implicitly suggests is that “a guy in a super nice hummer” means that he is a wealthy person, in addition to prior arguments describing such a vehicle as “nice and shiny and expensive looking.” Thus, DC's strategy here is to leverage rape myths regarding the notion that rape allegations are fabricated for monetary gain (Smith 2018) to undermine JD2's credibility.

Moreover, here in Extract 3, the complainant, previously identified as homeless and living on the street, is depicted as approaching a man in an expensive car. The conditions of being homeless and approaching someone from the street can be interpreted as a natural predicate and a category‐bound activity, respectively, of sex workers, as for Jane Doe 1. Thus, DC's association of JD2 with sex work endeavors to divest the complainant of her victim status, invoking the aforementioned rape myth that “people working in prostitution cannot be raped” (Silver et al. 2015).

In response to this attack to her credibility, JD2's sole resource is to account for her “hearable morally wrong” actions (“because,” line 4) by describing the accused as “like a friend,” thereby implying familiarity and trustworthiness rather than financial interest, as explicitly stated by JD2 (“not because of the hummer”). Furthermore, JD2 utters the word “hummer” through a laughter, exactly as DC has done in Extract 1, 4 about “running,” thus recurring to resources of courtroom interaction typically available only to attorneys. In the same way as before, irony is produced, further weakening the relevance of money and, more in general, the defendant's wealth (Clift 1999).

The next excerpt deepens this strategy by shifting from direct accusations to insinuations framed through third‐party perspectives. This rhetorical move enables the defense to imply moral judgment without stating it explicitly, maintaining a veneer of objectivity while perpetuating gendered stereotypes.

Extract 4 **“CA v. Kellen Winslow II”, Jane Doe 2 (JD2) cross‐examination by defence counsel (DC).**

1
 1 DC   Is it fair for me to say, (.) that your‐**the people that are**
 2      **on the streets? (.) would see a >very nice hummer?< (.) come**
 3      **up? (.) and you walk up and talk to the person in the HUmmer?**
 4      (.) >i mean that's fair for me to say isn't it,<=
 5 JD2   [=I mean]         [I: ]
 6 PA    [=Objec ]tion it lacks foundati[on ]and calls for speculation=
 7 J    =JOverruled go ah[ea]d=
 8 JD2            [I]−
 9 JD2   =**I didn't think much of it. (.) .h i don't chare if my**
10      **fri(.)end .h is down in the street, with no food or no**
11      **money, (.) .h I don't chare they've thons of money. .hh**
12      if they (stree)‐treat me respectfully, (.) i thalk to 'em.=

DC's turn, starting in line 1, opens with a polarized question, designed to solicit a “yes.” Furthermore, in this specific instance, DC does not directly ask JD2 about her actions. Instead, he employs an “epistemological filter” (Matoesian 1993) by questioning what others presumably observed her doing (“the people on the streets would see,” lines 1 and 2). This tactic mimics how courtroom members perceived JD2's actions through the lens of DC's questioning, invoking once again the implication that JD2 was acting more like a sex worker than a potential rape victim, who, according to prevailing stereotypes, should be passive, innocent, and emotionally fragile to be deemed credible (Christie 1986).

While interactional norms would compel JD2 to agree, doing so would imply acceptance of the accusation that she is not a legitimate victim or not an “ideal victim” victim, as for JD1. Consequently, she begins her turn by offering an alternative description (“I mean,” line 5). A brief overlap occurs as the Prosecuting Attorney (PA) raises an objection, which the Judge (J) overrules. JD2 then resumes her testimony on line 9, as if that interruption never happened. Similar to the prior Extract, JD2's rhetoric here reflects common strategies employed only by attorneys, by the use of “poetic repetition” (Matoesian 1995) and a three‐part list (Hutchby and Wooffit 1998). Both are conversational techniques used to strengthen one's position. JD2's repetition of “I didn't think/I don't care” combined with the list “[to be] down in the streets, with no food or no money” (lines 9 and 10) serves to protect her credibility. Additionally, to justify her speaking with the defendant, JD2 rejects any implication of interest in his wealth (“If they treat me respectfully, I talk to them,” line 12).

In summary, Jane Doe 2's strategy to resist secondary victimization involves providing alternative accounts for her actions. Though the mechanisms to avoid self‐blame and accusations have been noted previously (Atkinson and Drew 1979; Komter 1994), what is striking in trials involving gender‐based violence is the presence of a “double bind” (Matoesian 1993): the moral dimension of being a woman and stereotypical patriarchal values, manifested as rape myths and gendered stereotypes, are used as evidence to challenge a witness's credibility. The defendant, though, is not subjected to the same moral judgment about his identity and his past and/or current intimate life.

## 5 Managing the “Double Bind”

In conclusion, analyzing courtroom transcripts offers compelling insights into how secondary victimization takes place through talk in interaction, revealing the pervasive influence of rape myths in trials for gender‐based violence. On the one hand, in fact, it emerges that the defense counsel's strategy is deeply infused by such stereotypes. On the other hand, nevertheless, the victims questioned have the need to put in place resisting strategies, being absent any external tool of defense.

In the case of Jane Doe 1, the victim's actions are associated by the defense to the ones of a sex worker. In both excerpts, the defendant's wealth and fame are invoked to imply either that the sexual act was consensual, thus discrediting the rape allegation in line with the myth that “sex workers cannot be raped” (Silver et al. 2015), or that no sexual encounter occurred. These implications are reinforced by underlying the fact that, for example, Jane Doe 1 did not try to protect herself in anyway when the crime happened: rather, she only run when she was out of peril (Extract 1); and she initially did not report the crime, and later she did not cooperate with the investigations (Extract 2). Such behavior is mentioned by the counsel to solicit the rape myths that see rape victims fighting their assailant to not suffer the crime (Ellison and Munro 2013) and that delay in reporting is suspicious (Carr et al. 2014), respectively. Other themes, not exemplified in the present work, that the defense inquired about where about the clothes Jane Doe 1 was wearing (namely, “white leather pants” that the complainant constantly repaired as “white pants”), the fact that she only worried about her backpack—in a similar fashion as attorney Roy Black in the William Kennedy Smith trial, who inquired about the victim's prime interest of recovering her shoes from the scene of the crime (Matoesian 1993)—and that there was no use of force by the defendant in undressing Jane Doe 1 who, actually, undressed herself. The use of such themes to cast doubt on the allegations are too rooted in rape myths (Smith 2018) and ignore the role of fear and trauma on the victim's actions. Perceiving the attacks on her credibility, and the implicit accusation of having fabricated the complaint, Jane Doe 1 tries to resist using the only interactional resources she has being a witness: offering alternative descriptions of what was happening as accounts for her actions, trying her best to not agree to such accusations.

Similarly, Jane Doe 2 strategically placed alternative accounts facing such questions, offering explanations for her actions in a way to counter the accusations. Forced to being associated as well with being a sex worker, she offered descriptions of herself as a morally upright person interested in being respected, rather than wealth and money. Differently than Jane Doe 1, though, she did so by using the same interactional resources that the defense council showed in Jane Doe 1's cross‐examination (e.g., irony, poetic repetitions, etc.).

These findings resonate with recent research emphasizing the enduring influence of rape myths in legal contexts. Hudspith et al. (2024) show that while educational interventions aimed at reducing rape myth acceptance can enhance juror impartiality, their effects remain inconsistent and limited by methodological constraints. Similarly, Daly et al. (2022) caution against prematurely dismissing the role of rape myths in jury reasoning, highlighting how subtle and persistent biases may still shape deliberative processes. Together, these studies reinforce the conclusions drawn here: secondary victimization is not only enacted through defense discourse but also embedded in wider institutional and cultural practices that continue to legitimize gendered assumptions about credibility and consent. Further supporting these observations, Willmott and Hudspith (2024) provide a comprehensive synthesis of empirical evidence and stakeholder perspectives on rape myth bias in jury trials. Their analysis confirms that rape myths continue to shape juror reasoning and trial outcomes, despite ongoing reforms, and highlights growing professional recognition of this bias among lawyers, judges, and victim advocates. Crucially, they identify targeted educational interventions, such as enhanced judicial directions and pre‐trial juror training, as the most pragmatic and evidence‐based strategies for mitigating such prejudice.

Building on this, the present work makes an important contribution both topically and methodologically. Empirically, it extends research on the interactional reproduction and resistance of rape myths in courtroom discourse. Methodologically, it demonstrates the value of Ethnomethodology and Conversation Analysis (EMCA), a framework still underutilized in legal‐linguistic research, for uncovering the sequential organization of institutional talk that produces or challenges gendered harm. While grounded in a tradition of EMCA courtroom studies (Atkinson and Drew 1979; Matoesian 1993; Ehrlich 2001), this analysis advances that field by integrating a gender‐sensitive, intersectional focus, thereby bridging the gap between micro‐level interactional analysis and broader socio‐legal understandings of justice.

Thus, these findings highlight a critical need (Council of Europe 2012): both prosecution and defense counsels, as well as judges and jury members, must become more aware of rape myths and gendered stereotypes. Specifically, defense attorneys need to understand how to avoid invoking these concepts and, crucially, prosecution and judges need to understand how to protect victims from secondary victimization. Therefore, future research could investigate the impact of judicial and legal training on reducing secondary victimization in courtrooms; and furthermore expand the study of secondary victimization in sexual crimes trials with LGBTQ+ victims, and female‐to‐male rape, two areas still under researched but likewise heavily influenced by heteronormative and patriarchal stereotypes and values.

## Author Contributions

The author takes full responsibility for this article.

## Funding

The author has nothing to report.

## Ethics Statement

Research conducted with ethical approval of the Department of Political Sciences—University of Perugia, granting anonymity of all vulnerable parties. No ethical ID available.

## Conflicts of Interest

The author declares no conflicts of interest.

## Data Availability

Video recordings of the data available in full on YouTube. Transcripts are available at reasonable requests from the author.

## References

[bsl70025-bib-0001] Aina, O. A. , and A. E. Anowu . 2023. “Some Pragmatic Points of Description of Conducive Questioning in Courtroom Interrogation.” Journal of Universal Language 24, no. 2: 1–30. 10.22425/jul.2023.24.2.1.

[bsl70025-bib-0002] Atkinson, M. , and P. Drew . 1979. Order in Court: The Organisation of Verbal Interaction in Judicial Settings. Macmillan.

[bsl70025-bib-0003] Bernau, A. 2007. Virgins: A Cultural History. Granta Books.

[bsl70025-bib-0004] Bohner, G. , F. Eyssel , A. Pina , F. Siebler , and G. T. Viki . 2009. “Rape Myth Acceptance: Cognitive, Affective, and Behavioral Effects of Beliefs That Blame the Victim and Exonerate the Perpetrator.” In Rape: Challenging Contemporary Thinking, edited by M. A. H. Horvath and J. M. Brown . Willan Publishing.

[bsl70025-bib-0005] Brescoll, V. L. 2016. “Leading With Their Hearts? How Gender Stereotypes of Emotion Lead to Biased Evaluations of Female Leaders.” Leadership Quarterly 27, no. 3: 415–428. 10.1016/j.leaqua.2016.02.005.

[bsl70025-bib-0006] Brownmiller, S. 1975. Against Our Will: Men, Women and Rape. Bantam Books.

[bsl70025-bib-0007] Burrowes, N. 2013. Responding to the Challenge of Rape Myths in Court: A Guide for Prosecutors. NB Research.

[bsl70025-bib-0008] Carr, M. , A. J. Thomas , D. Atwood , A. Muhar , K. Jarvis , and S. S. Wewerka . 2014. “Debunking Three Rape Myths.” Journal of Forensic Nursing 10, no. 4: 217–225. 10.1097/jfn.0000000000000044. 25411813

[bsl70025-bib-0009] Christie, N. 1986. “The Ideal Victim.” In From Crime Policy to Victim Policy (17–30), edited by E. A. Fattah . MacMillan Press.

[bsl70025-bib-0010] Clayman, S. E. , and D. W. Maynard . 1995. “Ethnomethodology and Conversation Analysis.” In Situated Order: Studies in the Social Organisation of Talk and Embodied Activities, edited by P. ten Have and G. Psathas . University Press of America.

[bsl70025-bib-0011] Clift, R. 1999. “Irony in Conversation.” Language in Society 28, no. 4: 523–553. 10.1017/S0047404599004029.

[bsl70025-bib-0012] Conley, J. M. , and W. O’Barr . 2005. Just Words: Language and Power (2nd Edition). University of Chicago Press.

[bsl70025-bib-0013] Cora Garcia, A. 2023. An Introduction to Interaction: Understanding Talk in the Workplace and Everyday Life. 1st ed. Bloomsbury. 2013.

[bsl70025-bib-0014] Council of Europe, 2012 Council of Europe . 2012. *Convention on Preventing and Combating Violence Against Women and Domestic Violence (Istanbul Convention)*.

[bsl70025-bib-0015] Craig, E. 2021. Putting Trials on Trial: Sexual Assault and the Failure of the Legal Profession. McGill‐Queen's University Press.

[bsl70025-bib-0016] Crown Prosecution Service (CPS) . 2013. Charging Perverting the Course of Justice and Wasting Police Time in Cases Involving Allegedly False Rape and Domestica Violence Allegations. Crown Prosecution Service Equality & Diversity Unit.

[bsl70025-bib-0017] Daly, E. , O. Smith , H. Bows , et al. 2022. “Myths About Myths? A Commentary on Thomas (2020) and the Question of Jury Rape Myth Acceptance.” Journal of Gender‐Based Violence 7, no. 1: 189–200. 10.1332/239868021x16371459419254.

[bsl70025-bib-0018] Dinos, S. , N. Burrowes , K. Hammond , and C. Cunliffe . 2015. “A Systematic Review of Juries' Assessment of Rape Victims: Do Rape Myths Impact on Juror Decision‐Making?” International Journal of Law, Crime and Justice 43, no. 1: 36–49. 10.1016/j.ijlcj.2014.07.001.

[bsl70025-bib-0019] Drew, P. 1979. “The Production of Justifications and Excuses by Witnesses in Cross‐Examination.” In Order in Court, edited by J. M. Atkinson and P. Drew , 136–187. Humanities Press.

[bsl70025-bib-0020] Drew, P. 1998. “Complaints About Transgressions and Misconduct.” Research on Language and Social Interaction 31, no. 3–4: 295–325. 10.1080/08351813.1998.9683595.

[bsl70025-bib-0021] Dupret, B. , and M. Lynch . 2015. Law at Work: Studies in Legal Ethnomethods. Oxford University Press.

[bsl70025-bib-0022] Ehrlich, S. 2001. Representing Rape. Language and Sexual Consent. Routledge.

[bsl70025-bib-0023] Ellison, L. , and V. E. Munro . 2009. “Reacting to Rape: Exploring Mock Jurors’ Assessments of Complainant Credibility.” British Journal of Criminology 49, no. 2: 202–219. 10.1093/bjc/azn077.

[bsl70025-bib-0024] Ellison, L. , and V. E. Munro . 2013. “Better the Devil You Know? ‘Real Rape’ Stereotypes and the Relevance of a Previous Relationship in (Mock) Juror Deliberations.” International Journal of Evidence and Proof 17, no. 4: 299–322. 10.1350/ijep.2013.17.4.433.

[bsl70025-bib-0025] Estrich, S. 1976. Real Rape: How the Legal System Victimises Women Who Say No. Harvard University Press.

[bsl70025-bib-0026] Fenton, Z. 1998. “Domestic Violence in Black and White: Racialised Gender Stereotypes in Gender Violence.” Columbia Journal of Gender and Law 8, no. 1: 1–65. 10.7916/cjgl.v8i1.2400.

[bsl70025-bib-0027] Fitzgerald, R. , and W. Housley , eds. 2015. Advances in Membership Categorisation Analysis. SAGE Publications Ltd. 10.4135/9781473917873.

[bsl70025-bib-0028] Garfinkel, H. 1967. Studies in Ethnomethodology. Prentice Hall.

[bsl70025-bib-0029] Goodwin, C. 1994. “Professional Vision.” American Anthropologist 96, no. 3: 606–633. 10.1525/aa.1994.96.3.02a00100.

[bsl70025-bib-0030] Grilli, L. 1998. Il Dibattimento Penale. CEDAM.

[bsl70025-bib-0031] Gunby, C. , and A. Carline . 2020. “The Emotional Particulars of Working on Rape Cases: Doing Dirty Work, Managing Emotional Dirt and Conceptualizing ‘Tempered Indifference’.” British Journal of Criminology 60, no. 2: azz054. 10.1093/bjc/azz054.

[bsl70025-bib-0032] Heritage, J. 2004. “Conversation Analysis and Institutional Talk: Analysing Data.” In Qualitative Research: Theory, Method and Practice, edited by D. Silverman , 222–245. Sage Publications.

[bsl70025-bib-0033] Heritage, J. , and S. Clayman . 2010. Talk in Action: Interactions, Identities, and Institutions. Blackwell.

[bsl70025-bib-0034] Hester, S. , and P. Eglin . 1997. “Membership Categorization Analysis: An Introduction.” In Culture in Action (1–24), edited by S. Hester and P. Eglin , University Press of America.

[bsl70025-bib-0035] Hildebrand‐Edgar, N. , and S. Ehrlich . 2017. “‘She was Quite Capable of Asserting Herself’: Powerful Speech Styles and Assessments of Credibility in a Sexual Assault Trial.” Language and Law/Linguagem e Direito 4, no. 2: 89–107. https://ojs.letras.up.pt/index.php/LLLD/article/view/3285.

[bsl70025-bib-0036] Hudspith, L. F. , N. Wager , D. Willmott , and B. Gallagher . 2024. “The Impact of Rape Myth Education on Jury Decision‐Making: A Systematic Review.” Trauma, Violence, & Abuse 25, no. 5: 4062–4077. 10.1177/15248380241270082. PMC11545151 39268945

[bsl70025-bib-0037] Hutchby, I. , and R. Wooffit . 1998. Conversation Analysis. Polity Press.

[bsl70025-bib-0038] Italian Parliamentary Commission of Inquiry on Femicide and All Forms of Gender‐Based Violence, 2018 Italian Parliamentary Commission of Inquiry on Femicide and All Forms of Gender‐Based Violence . 2018. *La vittimizzazione secondaria delle donne che subiscono violenza e dei loro figli nei procedimenti che disciplinano l’affidamento e la responsabilità genitoriale*.

[bsl70025-bib-0039] Komter, M. 1994. “Accusations and Defences in Courtroom Interaction.” Discourse & Society 5, no. 2: 165–187. 10.1177/0957926594005002002.

[bsl70025-bib-0040] Lees, S. 1997. Carnal Knowledge: Rape on Trial. Women’s Press.

[bsl70025-bib-0041] Madigan, L. , and N. C. Gamble . 1991. The Second Rape: Society’s Continued Betrayal of the Victim. Lexington Books.

[bsl70025-bib-0042] Martin, P. Y. , and R. M. Powell . 1994. “Accounting for the ‘Second Assault’: Legal Organizations’ Framing of Rape Victims.” Law & Social Inquiry 19, no. 4: 853–890. 10.1111/j.1747-4469.1994.tb00942.x.

[bsl70025-bib-0043] Matoesian, G. 1993. Reproducing Rape. University of Chicago Press.

[bsl70025-bib-0044] Matoesian, G. 1995. “Language, Law and Society. Policy Implication in the Kennedy‐Smith Rape Trial.” Law & Society Review 29, no. 4: 669–701. 10.2307/3053918.

[bsl70025-bib-0045] Maynard, D. W. , and J. F. Manzo . 1993. “On the Sociology of Justice: Theoretical Notes From an Actual Jury Deliberation.” Sociological Theory 11, no. 2: 171–193. 10.2307/202141.

[bsl70025-bib-0046] Pemberton, A. , and E. Mulder . 2023. “Bringing Injustice Back in: Secondary Victimization as Epistemic Injustice.” Criminology and Criminal Justice: 1181–1200. 10.1177/17488958231181345.

[bsl70025-bib-0047] Pomerantz, A. 1984. “Agreeing and Disagreeing With Assessments: Some Features of Preferred/Dipreferred Turn Shapes.” In Structures of Social Action: Studies in Conversational Analysis, edited by J. M. Atkinson and J. Heritage , 57–101. Cambridge University Press.

[bsl70025-bib-0048] Pomerantz, A. 1988. “Offering a Candidate Answer: An Information Seeking Strategy.” Communications Monographs 55, no. 4: 360–373. 10.1080/03637758809376177.

[bsl70025-bib-0049] Raymond, G. 2003. “Grammar and Social Organization: Yes/No Interrogatives and the Structure of Responding.” American Sociological Review 68, no. 6: 939–967. 10.2307/1519752.

[bsl70025-bib-0050] Richardson, E. 2024. “Increasing Access and Transparency: Evaluating Transcript Provision for Rape Victim‐Survivors in Scottish Legal Proceedings.” Journal of Criminal Psychology 14, no. 4: 428–431. 10.1108/JCP-03-2024-0026.

[bsl70025-bib-0051] Richardson, E. , L. Jenkins , and D. Willmott . 2025. “Rape Myths, Jury Deliberations, and Conversation Analysis: Examining Conversational Practices Used to Undermine Rape Complaints Within (Mock) Jury Deliberations.” Journal of Criminal Justice 99, no. 2025: 102461. ISSN 0047‐2352. 10.1016/j.jcrimjus.2025.102461.

[bsl70025-bib-0052] Rose, M. R. , J. Nadler , and J. Clark . 2006. “Appropriately Upset? Emotion Norms and Perceptions of Crime Victims.” Law and Human Behavior 30, no. 2: 203–219. 10.1007/s10979-006-9030-3. 16786407

[bsl70025-bib-0053] Sacks, H. 1972. “On the Analyzability of Stories by Children.” In Directions in Sociolinguistics: The Ethnography of Communication, edited by J. J. Gumperz and D. Hymes . Rinehart and Winston.

[bsl70025-bib-0054] Sacks, H. 1992. In Lectures on Conversation, Vol. 1, edited by G. Jefferson . Blackwell.

[bsl70025-bib-0055] Sacks, H. , E. A. Schegloff , and G. Jefferson . 1974. “A Simplest Systematics for the Organization of Turn‐Taking for Conversation.” Language 50, no. 4: 696. 10.2307/412243.

[bsl70025-bib-0056] Schegloff, E. A. , and H. Sacks . 1973. “Opening up Closings.” Semiotica 8, no. 4: 289–327. 10.1515/semi.1973.8.4.289.

[bsl70025-bib-0057] Schneider, D. J. 2004. The Psychology of Stereotyping. Guilford Press.

[bsl70025-bib-0058] Shields, S. A. 2002. Speaking From the Heart: Gender and the Social Meaning of Emotion. Cambridge University Press.

[bsl70025-bib-0059] Shoham, S. G. , P. Knepper , and M. Kett . 2010. International Handbook of Victimology. CRC Press.

[bsl70025-bib-0060] Silver, K. , G. Karakurt , and S. Boysen . 2015. “Predicting Prosocial Behaviour Toward Sex‐Trafficked Persons: The Roles of Empathy, Belief in a Just World, and Attitudes Toward Prostitution.” Journal of Aggression, Maltreatment & Trauma 24, no. 8: 932–954. 10.1080/10926771.2015.1070231.

[bsl70025-bib-0061] Smith, O. 2018. Rape Trials in England and Wales: Observing Justice and Rethinking Rape Myths. Palgrave Macmillan.

[bsl70025-bib-0062] Smith, O. , and T. Skinner . 2017. “How Rape Myths Are Used and Challenged in Rape and Sexual Assault Trials.” Social & Legal Studies 26, no. 4: 441–466. 10.1177/0964663916680130.

[bsl70025-bib-0063] Stokoe, E. H. 2003. “Mothers, Single Women and Sluts: Gender, Morality and Membership Categorization in Neighbour Disputes.” Feminism & Psychology 13, no. 3: 317–344. 10.1177/0959353503013003006.

[bsl70025-bib-0064] Symonds, M. 1980. “Victim Responses to Terror.” Annals of the New York Academy of Sciences 347, no. 1: 129–136. 10.1111/j.1749-6632.1980.tb21262.x. 6930892

[bsl70025-bib-0065] Taslitz, A. 1999. Rape and the Culture of the Courtroom. New York University Press.

[bsl70025-bib-0066] Temkin, J. , and B. Krahé . 2008. Sexual Assault and the Justice Gap: A Question of Attitude. Hart Publications.

[bsl70025-bib-0067] Tennent, E. , and E. Richardson . 2025. “Domestic Violence and the Spatial‐Moral Order of Home in Calls to Police.” Journal of Criminal Psychology 15, no. 7: 31–45. 10.1108/JCP-12-2024-0136.

[bsl70025-bib-0068] Travers, M. , and J. F. Manzo . 1997. Law in Action: Ethnomethodological and Conversation Analytic Approaches to Law. 1st ed. Routledge. 10.4324/9781315250663.

[bsl70025-bib-0069] Wemmers, J.‐A. 2013. “Victims’ Experiences in the Criminal Justice System and Their Recovery From Crime.” International Review of Victimology 19, no. 3: 221–233. 10.1177/0269758013492755.

[bsl70025-bib-0070] Williams, J. 1984. “Secondary Victimization: Confronting Public Attitudes About Rape.” Victimology Volume 9, no. 1: 66–81.

[bsl70025-bib-0071] Willmott, D. , and L. Hudspith . 2024. “Jury Trials and Rape Myth Bias: Exploring the Research Evidence, Stakeholder Perspectives and Effective Solutions.” In Contemporary Challenges in the Jury System A Comparative Perspective. 1st ed., edited by N. Monaghan . Routledge.

